# Association of negative symptom dimensions with sleep efficiency in schizophrenia patients with predominant negative symptoms

**DOI:** 10.1192/j.eurpsy.2025.2238

**Published:** 2025-08-26

**Authors:** Ž. Bajić, V. Grošić, K. Matić, I. Š. Filipčić, P. F. Grošić, K. Bosak, I. Filipčić

**Affiliations:** 1 Psychiatric Clinic Sveti Ivan; 2 University Hospital Centre Zagreb, Zagreb; 3 Faculty of Dental Medicine and Health, Josip Juraj Strossmayer University of Osijek, Osijek, Croatia

## Abstract

**Introduction:**

Negative symptoms, such as avolition, blunted affect, or alogia, contribute to functional disability and reduced quality of life in schizophrenia. Patients with predominant negative symptoms and minimal positive symptoms represent a distinct subgroup requiring tailored therapeutic strategies. Sleep disturbances, particularly reduced sleep efficiency, are commonly reported in this population and may exacerbate the severity of negative symptoms. Understanding the differential impact of specific negative symptoms on sleep efficiency could inform individualized approaches for improving otucomes.

**Objectives:**

To explore associations between distinct dimensions of negative symptoms and sleep efficiency in schizophrenia patients with predominant negative symptoms and low positive symptoms.

**Methods:**

This analysis used baseline data from a randomized, sham-controlled trial on the efficacy of transcranial magnetic stimulation in schizophrenia, conducted between 2000 and 2023. The study included patients with PANSS negative subscale score > 24 and PANSS positive subscale score < 20. The outcome variable was the sleep efficiency subscale of the Pittsburgh Sleep Quality Index. Independent variables were the five SANS dimensions: blunted affect, alogia, avolition(/apathy, anhedonia/asociality, and attention impairment. Quantile regression was used to assess associations, and robust standard errors were applied.

**Results:**

We included 76 patients (median age 36 years, 33% women). Alogia was positively associated with sleep efficiency (β = 4.41, p = 0.040), while avolition (β = -3.61, p = 0.014) and attention impairment (β = -4.12, p = 0.041) were negatively associated. Blunted affect and anhedonia/asociality were not significantly associated with sleep efficiency.

**Conclusions:**

Distinct negative symptom dimensions show differential associations with sleep efficiency in schizophrenia patients with predominant negative symptoms. Alogia’s association with better sleep efficiency may reflect reduced mental arousal and fewer ruminative thoughts before sleep. Conversely, avolition and impaired attention may worsen sleep through increased inactivity and fragmented sleep patterns. These findings suggest that targeted therapeutic interventions may be necessary to optimize sleep and overall clinical management in this subgroup of patients. Further studies are needed to explore underlying mechanisms and clinical implications of the presented associations.

**Disclosure of Interest:**

None Declared

